# Supplementary material for: CSA Antisense Targeting Enhances Anticancer Drug Sensitivity in Breast Cancer Cells, including the Triple-Negative Subtype
Source: Cancers (Basel). 2022 Mar 26;14(7):1687. doi: 10.3390/cancers14071687 (PMC8997023; doi:10.3390/cancers14071687)

Supplementary Materials File S1

Figure 1 panel A.

	CSA	ACTB	CSA/ACTB
**MCF-10A**	7087,924	25203,15	0,281231683
**MCF-7**	19732,94	26595,18	0,741974175
**MDA-MB231**	35383,69	31527,11	1,122325746
**T47D**	33789,93	23916,35	1,412837954

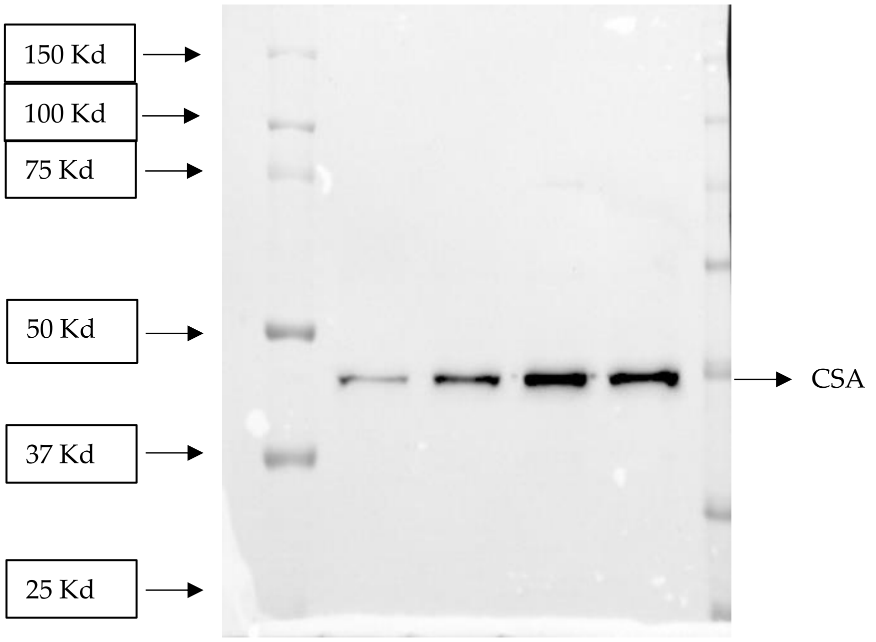

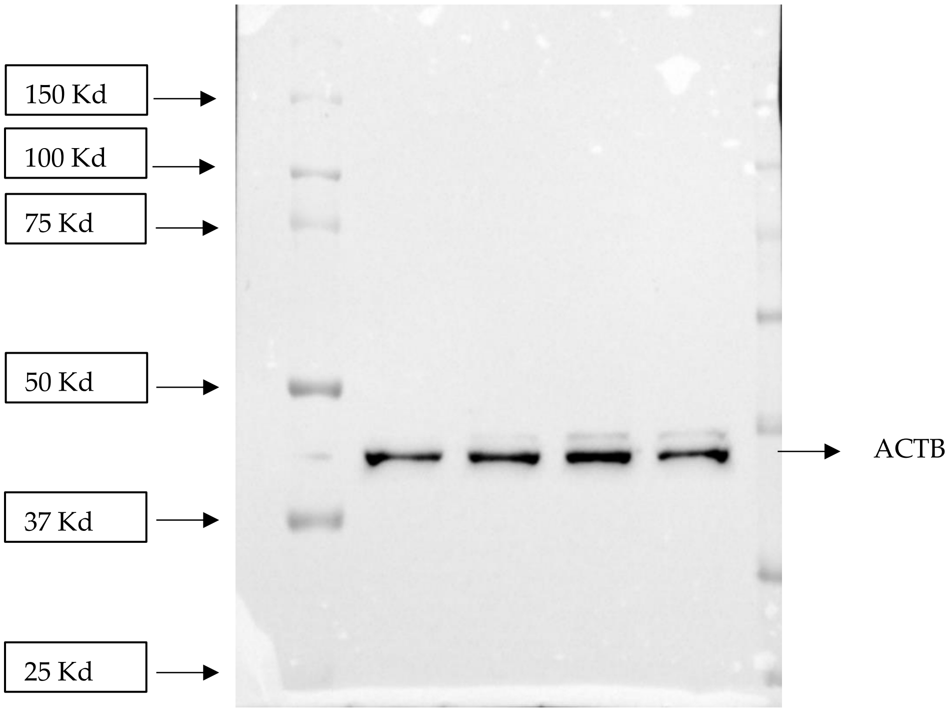

Figure 5 panel A

	Casp.3 cleaved	α-tubulin	CSA/ACTB
**Medium**	696,506	31972,9	0,021784262
**ASO**	595,420	26483,51	0,022482818
**PTX**	3525,314	35874,12	0,098269003
**ASO+PTX**	10527,675	42102,66	0,25004777
**OXA**	1943,77	26839,44	0,072422146
**ASO+OXA**	5039,606	23085,13	0,218305309

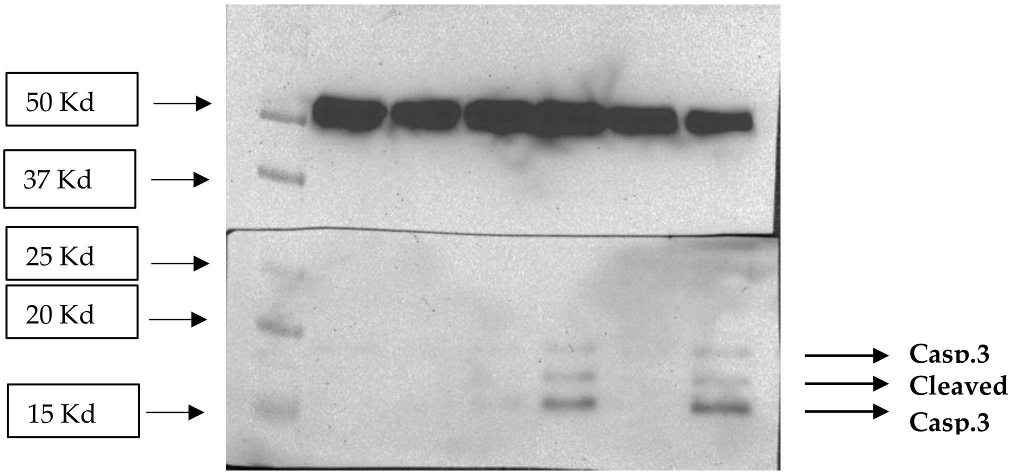

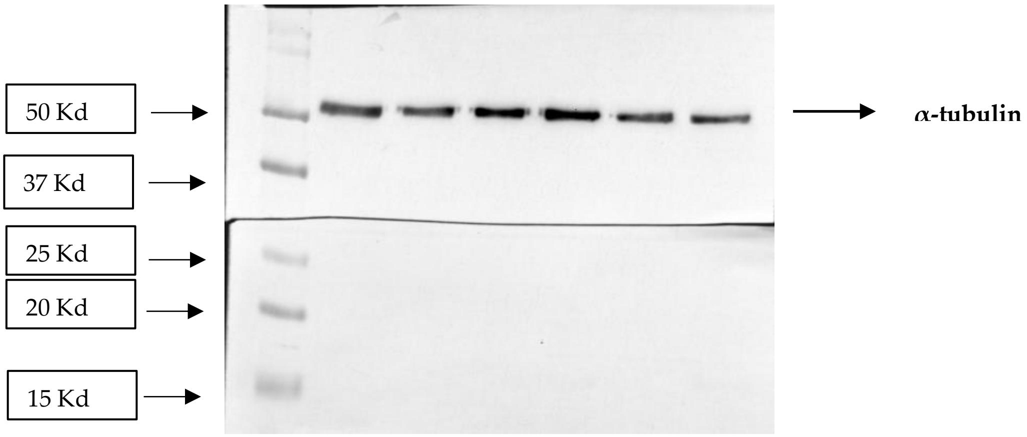

Supplement: Supplementary file 1 [file cancers-14-01687-s001.zip › cancers-1627772-supplementary.pdf]
